# Mania Induced by Epidural Steroid Injection in an Elderly Female With No Psychiatric History

**DOI:** 10.7759/cureus.12594

**Published:** 2021-01-09

**Authors:** Pauline Chen, Kelvin Tran, Tessy Korah

**Affiliations:** 1 Psychiatry, University of Florida, Gainesville, USA; 2 Psychiatry, University of Florida, Gainsville, USA

**Keywords:** substance induced disorders, psychosis, epidural steroid injection, substance-induced disorders, hypomania, geriatric psychiatry, geriatric medicine, altered mental state, altered mental status evaluation, psychiatry of old age

## Abstract

The psychiatric risks of epidural steroid injections for chronic pain in a geriatric patient with no prior psychiatric history are presented here. A 76-year old Caucasian female presented to the emergency department with her family for an inability to sleep, confusion, and behavioral outbursts. The mood instability and psychosis were reported as having started a week after her third epidural steroid injection for low-back pain associated with a prior fall. After 12 days of mixed treatment outcomes and increasing paranoia without any localized neurological findings, the patient was transferred to the geriatric psychiatry unit. Upon admission to the inpatient unit, she was loud, grandiose, verbally aggressive, unable to sleep, hyper-religious, paranoid, and identified her husband and daughter as demons. The patient was started on risperidone and valproic acid for the management of psychosis and manic symptoms. Hyper-religiosity and paranoia greatly improved within a week, though the patient remained very talkative and tangential, with a disorganized thought process. Valproic acid was titrated to 500 mg twice a day, yielding a level of 56.2 ug/ml, accompanied by improvement to mild talkativeness and circumstantiality. She was able to interact appropriately, with minimal lorazepam requirement, and discharged with a linear thought process and absence of psychosis. On outpatient follow up, there were minimal residual mania and no recurrence of psychosis, allowing her to be weaned off valproic acid and to discontinue risperidone. Two months later, symptoms resolved completely. The persistence of this patient’s psychosis for nearly one month, and mania for about three months, underscores the importance of careful risk-benefit analysis before initiating epidural steroids. This is particularly important in elderly patients who may be more susceptible to psychiatric adverse effects that can outlast any analgesic benefits.

## Introduction

About 5% of steroid-treated patients develop severe psychiatric reactions with predominately mood-related symptoms, including mania, psychosis, and delirium (28%, 14%, and 10% of the patients, respectively) [1]. While these symptoms may be severe or debilitating, they are typically short-lasting with a mean duration of psychiatric symptoms of around 21-22 days [1]. However, there are cases documented where patients have psychiatric symptoms of longer duration up to nearly six months [1-2]. This is concerning as patients may be at risk of having functionally impairing symptoms when a very commonly prescribed medication is delivered. Additionally, with the advancement of medications and technology, epidural steroid injections are becoming more common as interventional pain treatments with resultant concerns of corticosteroid-induced psychiatric outcomes [3-4]. However, there are limited studies evaluating corticosteroid-induced psychosis secondary to interventional pain procedures [5]. Especially unreported are geriatric psychosis cases associated with epidural steroid injections. Below is a case report detailing the adverse effect from a specific interventional pain procedure, an epidural steroid injection, in a senior adult.

This article was previously presented as a meeting abstract at the 2018 University of Florida College of Medicine (UF COM) Poster Session on February 19, 2018, at the Florida Psychiatric Society (FPS) meeting on April 14, 2018, and at the National Network of Depression Centers (NNDC) Conference on September 24, 2019.

## Case presentation

A 76-year-old Caucasian female with no psychiatric history presented to the emergency department with her family for a one-week history of unstable mood and psychosis following her third epidural steroid injection for low-back pain due to falls history. The chief complaint included altered mental status, agitation, and hallucinations. Table 1 shows the admission lab values that were significant.

**Table 1:** Significant Admission Labs

Serum Labs	
White Blood Cell Count	14,900/uL
Neutrophils Count	11,800/uL
Monocytes Count	1,000/uL
Sodium Level	128 mmol/L
Magnesium Level	1.6 mg/dL
Glucose Level	161 mg/dL
Ammonia Level	9 umol/L

The patient was treated for hyponatremia given low serum sodium and transferred to neurology for further workup. Magnetic resonance imaging (MRI) of the head and lumbar puncture were unremarkable. However, the initial electroencephalogram (EEG) was consistent with a partial seizure disorder and focal clinical status epilepticus in the right temporoparietal region. The patient was treated with phenytoin 300 mg daily. The patient also received quetiapine and later olanzapine for psychosis. No seizure events were captured on EEG study one week later. Table 2 lists the results of the initial and final EEG. The patient was also briefly treated with intravenous methylprednisolone for suspected autoimmune encephalitis without noted benefit. Despite treatment with antiepileptic medication, methylprednisolone, and sodium correction, the patient continued to exhibit symptoms of mania and psychosis and was transferred to the geriatric psychiatric inpatient unit 12 days after the initial presentation.

**Table 2:** EEG Results EEG: electroencephalogram

	EEG Results
Initial EEG	Multiple brief electrographic seizures were present during the recording, poorly localized to the right temporoparietal region, consistent with a partial seizure disorder and focal clinical status epilepticus manifesting as ictal/post-ictal psychosis & confusion.
Final EEG (one week later)	Abnormal EEG due to the presence of a mild degree of diffuse background slowing. No potentially epileptogenic activity or seizures were present during the recording.

Upon psychiatric admission, the patient continued to identify her husband and daughter as demons. Her mental status exam was significant for disheveled appearance, uncooperative behavior, loud and disorganized speech with loose associations, grandiose, hyper-religious, and paranoid thought content. The patient was placed on one-to-one observation with assault precautions. Risperidone was initiated to target psychosis and mood and titrated to 1 mg twice daily; intramuscular (IM) haloperidol and lorazepam were also utilized during an event of acute agitation and assault on a healthcare staff member. The patient tolerated the risperidone well, with noted less paranoia but was still emotionally labile; consequently, valproic acid (VPA) was initiated for mood stabilization and was titrated to 500 mg twice daily. VPA level was at a therapeutic plasma level of 56.2 ug/ml. The patient tolerated the VPA well, with noted less lability and grandiosity. Phenytoin was discontinued due to the development of a rash and replaced with lorazepam 1 mg three times a day for both the coverage of seizures and mood stability. Lorazepam was later tapered and discontinued by the time the patient was discharged. The patient eventually improved over two weeks in the geriatric psychiatric ward and was coherent by the time of discharge. The patient was discharged back home to family.

On outpatient follow up, there were minimal residual mania and an absence of psychosis. VPA and risperidone were tapered and discontinued. Two months later, symptoms resolved completely. Over the course of treatment, the patient’s psychosis lasted approximately one month while the mania persisted for about three months. Based on the patient's presentation, the patient had a steroid-induced bipolar and related disorder with psychotic features.

## Discussion

Comprehensive reviews published on the safety of cervical and lumbar epidural steroid injections stated that the most common adverse events are minor and self-limiting; the procedures are relatively safe [6-7]. However, as mania and psychosis have been demonstrated to be relatively common adverse complications with steroid use, there is a need for appropriate awareness and consideration of such complications [1-2]. This is especially important in the geriatric population. The report presented here demonstrates an example of such an event that was neither self-limited nor minor, leading to both prolonged significant functional impairment for the patient and psychosocial disturbance for the family. Regarding lumbar epidural steroid injections, meta-analysis has demonstrated modest analgesic benefit with no functional benefit at a median of three months [4]. Of note, the patient in our case report also took a similar length of time for her residual manic symptoms to remit. The persistence of this patient’s psychosis for nearly one month, and mania for about three months, underscores the importance of careful risk-benefit analysis before initiating epidural steroids.

Literature supports immediate procedural safety regarding epidural steroid injections for the elderly, as they appear to pose medium-term risks like any other form of corticosteroid treatment. However, many of these procedures are performed on geriatric patients who carry an increased risk of steroid-induced psychiatric complications that are not as emphasized. These psychiatric complications could then expose geriatric patients to psychotropic medications, which can further increase the risk of adverse effects. Interventional pain management providers and patients should consider the risk of prolonged, severe manic episodes with psychotic features as part of the informed consent process.

## Conclusions

This case exemplifies the importance of evaluating the risk vs. benefit of epidural steroid injections in the geriatric population. There is an emphasis on procedural safety with epidural steroid injections, but limited consideration for steroid-induced psychiatric complications. While steroid-induced mood symptoms, such as mania, psychosis, and delirium, are typically short-lasting, it is important to be aware that reactions may not be minor or short-lasting. As presented in this case, the patient developed psychosis that lasted about one month and mania for about three months, which required psychotropic medication for complete resolution of symptoms. In addition to safety concerns with an uncontrolled mood disorder, exposure to psychotropic medications further increases the risk of adverse effects in the elderly. Thus, it is important to consider the risk of prolonged, severe mood disorder episodes that may outlast the analgesic benefits of epidural steroid injections as part of the informed consent process for epidural steroid injections in the elderly, who may be more susceptible to psychiatric adverse effects.
